# Depression and anxiety in women with malignant ovarian germ cell (MOGCT) and sex cord stromal tumors (SCST): an analysis of the AGO-CORSETT database

**DOI:** 10.1007/s00404-022-06781-0

**Published:** 2022-09-21

**Authors:** M. Bossart, H. Plett, B. Krämer, E. Braicu, B. Czogalla, M. Klar, S. Singer, D. Mayr, A. Staebler, A. du Bois, S. Kommoss, T. Link, A. Burges, F. Heitz, M. Grube, F. Trillsch, P. Harter, P. Wimberger, P. Buderath, A. Hasenburg

**Affiliations:** 1 Department of Gynecology and Obstetrics, University Medical Center Freiburg, Freiburg im Breisgau, Germany; 2 Department of Gynecology and Obstetrics, St. Josefskrankenhaus Freiburg, Freiburg im Breisgau, Germany; 3 Department of Gynecology & Gynecologic Oncology, Ev. Kliniken Essen-Mitte (KEM), Essen, Germany; 4 Department of Women’s Health, Tuebingen University Hospital, Tuebingen, Germany; 5 Department of Gynecology, Charité Berlin, Campus Virchow Clinic, Berlin, Germany; 6 Department of Obstetrics and Gynecology, University Hospital, Ludwig- Maximilians- University Munich, Munich, Germany; 7 Institute of Medical Biostatistics, Epidemiology and Informatics, Division of Epidemiology and Health Care Research, University Medical Center Mainz, Mainz, Germany; 8 Institut of Pathology, Ludwig-Maximilians- University Munich, Munich, Germany; 9 Division of Gynecologic Pathology, Institute of Pathology and Neuropathology, University of Tuebingen, Tuebingen, Germany; 10 Department of Gynecology and Obstetrics, Technische Universität Dresden Dresden and National Center for Tumor Diseases (NCT/UCC), Dresden, Germany; 11 Department of Gynecology and Obstetrics, University Hospital Essen, Essen, Germany; 12 Department of Gynecology and Obstetrics, University Medical Center Mainz, Mainz, Germany

**Keywords:** Ovarian germ cell tumors, Sex cord stromal tumors, Fertility-sparing surgery, Depression, Anxiety, Pain

## Abstract

**Introduction:**

The intention of this study was to evaluate the level of anxiety and depression of malignant ovarian germ cell (MOGCT) and sex cord stromal tumors (SCST) survivors and to identify possible alterable cofactors.

**Methods:**

CORSETT was an observational, multicenter, mixed retrospective/prospective cohort study of the AGO Studygroup. Women who had been diagnosed with MOGCTs and SCSTs between 2001 and 2011 were asked to complete the Hospital Anxiety and Depression Scale (HADS) to evaluate distress. Predictors of distress (type of surgery, chemotherapy, time since diagnosis, recurrence, second tumor, pain) were investigated using multivariate linear regression analysis.

**Results:**

150 MOGCT and SCST patients with confirmed histological diagnosis completed the questionnaire median seven years after diagnosis. They had a HADS total score ≥ 13 indicating severe mental distress in 34% of cases. Patients after fertility-conserving surgery had lower probability of severe mental distress than those without fertility-conserving treatment (*β* = − 3.1, *p* = 0.04). Pain was associated with the level of distress in uni- and multivariate analysis (coef 0.1, *p* < 0.01, coef. Beta 0.5).

**Discussion:**

Severe mental distress was frequent in patients with MOGCT and SCST and the level of pain was associated with the level of distress. Fertility conserving therapy, however, was associated with less mental distress. Screening and treatment of pain and depression is required to improve mental well-being in survivors of MOGCT and SCST.

## What does this study add to the clinical work

**Table:** 

Depression and anxiety in women with MOGCT and SCST are frequent and related to the level of pain even years after the first diagnose. Fertility conserving surgery is associated with less depression and anxiety these young cancer survivors.	

## Purpose

Malignant ovarian germ cell (MOGCT) and sex cord stromal tumors (SCST) are rare ovarian neoplasms that, however, affect a disproportionally high number of young patients. In a population-based study, MOGCT comprised only 1.3% and SCST 1.6% of all ovarian malignancies in Europe in the time period of 2005–2009 [1]. In an analysis of the Danish cancer registry, the age-standardized incidence rate was 0.39 for MOGCT and 0.09 for SCST per 100,000 women-years in 2008–2016 [2]. Patients with MOGCT and SCST typically have, irrespective of stage, higher survival rates than patients with epithelial ovarian cancer [3]. As SCST and MOGCT mainly affect young women, long-term survivors are frequent and quality of life as well as fertility preservation during cancer treatment are relevant topics.

MOGCT represent about 75% of malignant ovarian tumors in children [4]. The most common types of MOGCT are dysgerminoma and immature teratoma, followed by yolk sac tumors and mixed germ cell tumors [5]. The treatment of MOGCT mainly comprises surgical tumor resection and chemotherapy. MOGCT are radiosensitive but radiotherapy is only indicated in selected cases as chemotherapy is very effective and less fertility reducing [6]. Fertility preserving surgery for women in childbearing age and with early disease is currently the gold standard [7]. According to the European Society of Medical Oncology (ESMO), a fertility-retaining approach is indicated for every woman with MOGCT wishing to retain her reproductive potential, even with advanced stage disease [8]. Adjuvant platinum-based chemotherapy with etoposide is considered necessary for patients beyond stage IA. Bleomycin or ifosfamid may be added in high risk constellations [6]. For all stages, 5-year disease free survival is 86% and 5-year overall survival 97% after fertility sparing surgery and adjuvant treatment [9].

SCST arise from the primitive sex cord and/or stromal cells of the gonads, including granulosa, theca, Sertoli, Leydig cells or fibroblasts. Middle-aged women are most commonly affected [10]. SCSTs often secret hormones which can lead to menstrual bleeding disorders or psychic alterations [10]. Surgery is required for staging and therapy. Granulosa cell tumors, Sertoli Leydig cell tumors (SLCTs) of G2/G3 grading and stroma cell tumors require surgical treatment analogous to ovarian cancer staging. Fertility-sparing surgery in young patients is an option. The benefit of adjuvant chemotherapy remains unclear, some patients with FIGO IC disease or residual tumor may benefit from platinum-containing protocols [6]. Five-year cause-specific survival is slightly lower than for MOGCT, at 88% across all stages [11].

In 2010, the global annual prevalence of depression in women was 5.5%, reporting a 1.7 fold greater incidence than in men [12]. Cancer increases the likelihood of anxiety and depression [13]. Significant psychological distress is common across all the stages of cancer as a life-threatening disease, with anxiety and depression representing its most typical manifestation [14]. Literature reports about 40% of ovarian cancer patients showing clinical levels of anxiety and depression [15, 16, 17]. The level of anxiety and depression in MOGCT and SCST patients is yet unknown.

Depressed cancer patients have a higher mortality than non-depressed patients and have lower health-related quality of life and lower adherence to cancer treatment [18, 19]. Therefore, national and international cancer societies recommend that all patients with cancer should be screened for depression throughout the trajectory of care [20]. Vulnerability to depression is increased across the menopause transition. Surgical menopause due to cancer treatment increases depression even further in ovarian cancer patients [21].

Cancer as well as surgical and adjuvant therapy can cause various patterns of pain in cancer patients. Pain prevalence is 55% during active cancer treatment and 39% after curative treatment. Moderate to severe pain was reported in 38% of all patients in a meta-analysis on various cancer types [22]. The intention of this study was to evaluate the level of anxiety and depression in MOGCT and SCST patients and to identify possible predictors with a special focus on fertility-sparing surgery and chemotherapy.

## Methods

The Current Ovarian geRm cell and SEx cord stromal Tumour Treatment strategies (CORSETT) study was an observational, multicenter, mixed retrospective and prospective cohort study of the Arbeitsgemeinschaft für Gynäkologische Onkologie (AGO) Studygroup. Women of any age who had been diagnosed with MOGCTs and SCSTs or dermoid cysts with immature/malignant somatic components between 2001 and 2011 were contacted by each center and consented to the study.

Patients with MOGCTs and SCSTs were asked to complete questionnaires to evaluate quality of life (Qol, EORTC QLQ-C30), anxiety and depression (HADS A and D). The Hospital Anxiety and Depression Scale (HADS) is a self-report questionnaire consisting of two subscales (HADS-A and HADS-D) designed to identify and quantify distress in physically ill patients [23]. The HADS consist of 14 items with a 4-point ordinal response format. Symptoms during the previous week are reported on a scale ranging from “0” (not at all) to “3” (most of the time). Scores range from zero to 42. High scores indicate higher levels of distress. It has been shown that the HADS total score has the best diagnostic accuracy for screening of depression in cancer patients at a threshold of 13 [15].

Pain was measured using the respective subscale of the European Organization for Research and Treatment of Cancer Quality of Life core questionnaire (EORTC QLQ-C30) [24]. Pain is assessed with item number 9 and 19. The raw score is estimated and standardized using a linear transformation with a score ranging from 0 to 100. A higher score represents a higher (“worse”) level of pain [25].

Before conducting the statistical analysis, a detailed analysis plan was developed by a team of clinicians and statisticians. The analyses included a description of the sample characteristics and potential predicting factors. The association of fertility-sparing surgery and of adjuvant chemotherapy with levels of distress was investigated using linear regression analyses separately for MOGCT and SCST while adjusting for age. In the full sample, we investigated whether the following variables were associated with distress level: type of surgery (laparoscopy, laparotomy, conversion, unknown; fertility sparing versus not), adjuvant chemotherapy (not received, received, unknown), time since diagnosis (in years), age at time of survey (< 50 years, 50–70 years, > 70 years), cohabitation (no partner, married or with partner, unknown), pain (continuous) using multivariate linear regression analysis.

## Results

### Sample description

The HADS was completed by 166 out of 290 patients (57.2%) who fulfilled the inclusion criteria. SCST was diagnosed in 104 of the responders, MOGCT in 46 and an unknown histology in 16 patients (they were excluded for this paper). Time since first diagnosis was median 7 years (mean 6 years). Patients were aged 17–86 years (mean 49, median 50 years).

MOGCT and SCST were mostly diagnosed in FIGO stage I (*n* = 125; 83%), 8 (5%) in FIGO II, 6 (4%) in FIGO III, one patient in FIGO IV. 10 patients (7%) had an unknown FIGO stage. Fertility-sparing surgery was performed in 85 (57%) patients. Laparoscopy was performed in 89 patients (59%), laparotomy in 54 patients (36%), in 4 patients; laparoscopy was converted to laparotomy within the surgery. Adjuvant chemotherapy was administered in 44 patients (29%). In 55 patients (37%), the tumor had relapsed. A second tumor was diagnosed in 13 patients (9%). Detailed data are shown in Table 1.

**Table 1:** Patients characteristics

	Number of patients	Total patients included		Only SCST		Only MOGCT	
		150		104		46	
		*N*	%	*N*	%	*N*	%
Histology	SCST granulosa cell	92	61%	92	88%		
	SCST sertoli leydig	12	8%	12	12%		
	OGCT mixed	13	9%			13	28%
	OGCT teratoma	14	9%			14	30%
	OGCT dysgerminoma	19	13%			19	41%
FIGO stage at diagnosis	I	125	83%	85	82%	40	87%
	II	8	5%	5	5%	3	7%
	III	6	4%	4	4%	2	4%
	IV	1	1%	1	1%	0	0%
	Unknown	10	7%	9	9%	1	2%
Surgical approach	Laparoscopy	89	59%	62	60%	27	59%
	Laparotomy	54	36%	38	37%	16	35%
	Conversion	4	3%	2	2%	2	4%
	Unknown	3	2%	2	2%	1	2%
	Non-Fertility sparing	58	39%	52	50%	6	13%
	Fertility sparing	85	57%	48	46%	37	80%
	Unknown	7	5%	4	4%	3	7%
Adjuvant chemotherapy	No	104	69%	84	81%	20	43%
	Yes	44	29%	18	17%	26	57%
	Unknown	2	1%	2	2%	0	0%
Recurrence	No	92	61%	53	51%	39	85%
	Yes	55	37%	48	46%	7	15%
	Unknown	3	2%	3	3%	0	0%
Second tumor	No	133	89%	89	86%	44	96%
	Yes	13	9%	12	12%	1	2%
	Unknown	4	3%	3	3%	1	2%

Most patients (*n* = 42, 91%) with MOGCT were younger than 50 years at survey (mean 34.1 years, median 31, range 17–70 years). The histology was mixed MOGCT in 13 (28%), teratoma in 14 (39%) and dysgerminoma in 19 (41%) patients. The median age of patients with dysgerminoma was 30 years, with teratoma 37.5 years and with mixed MOGCT 33 years. Fertility-sparing surgery was performed in 37 of the 46 patients (80%), 26 (57%) received adjuvant chemotherapy. Seven patients (15%) were diagnosed with recurrent disease (Table 1) and one (2%) had a second tumor.

Patients with SCST were on average 55.7 years old (median 56, range 17 to 86 years). Histologically, 92 (88%) had a granulosa cell tumor, 12 (12%) a Sertoli-Leydig cell tumor. The median age of patients with Granulosa cell tumors was 56 years and with Sertoli-Leydig cell tumors 45 years. Fertility-sparing surgery was performed in 48 patients (45%) and 18 (17%) received additional chemotherapy. In 47 (44%) patients a recurrence was diagnosed, 13 (9%) patients had a second tumor (Table 2).

**Table 2:** Association of distress with analyzed variables in ovarian germ cell and sex cord stromal tumors in univariate and multivariate analysis

		Univariate analysis				Multivariate analysis		
		Coef.	*P-value*	95% conf.	Interval	Coef.	*P-value*	Beta
Surgical approach	Laparoscopy	Ref				Ref		
	Laparotomy	0.6	*0.61*	− 1.7	2.8	0.1	*0.95*	0.0
	Conversion	6.4	*0.06*	− 0.3	13.1	0.8	*0.79*	0.0
	unknown	6.1	*0.12*	− 1.6	13.7	4.0	*0.27*	0.1
	Non-Fertility sparing	Ref				Ref		
	Fertility sparing	− 1.9	*0.11*	− 4.1	0.4	− 2.9	*0.04*	− 0.2
	unknown	− 2.6	*0.33*	− 7.9	2.6	− 0.7	*0.80*	0.0
Adjuvant Chemotherapy	No	Ref				Ref		
	Yes	0.8	*0.51*	− 1.6	3.2	0.8	*0.46*	0.1
	Unknown	− 2.2	*0.64*	− 11.7	7.2	− 3.5	*0.47*	− 0.1
Pain	Per point on pain scale	0.1	*<0.001*	0.1	0.2			
Time since diagnose	In years	0.2	*0.17*	− 0.1	0.4	0.1	0.46	0.1
Age at survey	<50 years	Ref				Ref		
	50-70 years	1.2	*0.33*	− 1.2	3.5	− 1.3	*0.34*	− 0.1
	>70 years	− 0.9	*0.61*	− 4.3	2.5	− 4.2	*0.04*	− 0.2
Recurrence	No	Ref				Ref		
	Yes	0.1	*0.93*	− 2.2	2.4	− 0.4	*0.78*	0.0
Second tumor	No	Ref				Ref		
	Yes	1.7	*0.39*	− 2.2	5.5	0.8	*0.67*	0.0

Distress was analyzed for the total cohort (*n* = 150). HADS total score was on average 10.5 (SD 6.7, range 0–32). 34% of patients had a score ≥ 13, indicating severe mental distress.

**Fig. 1:**
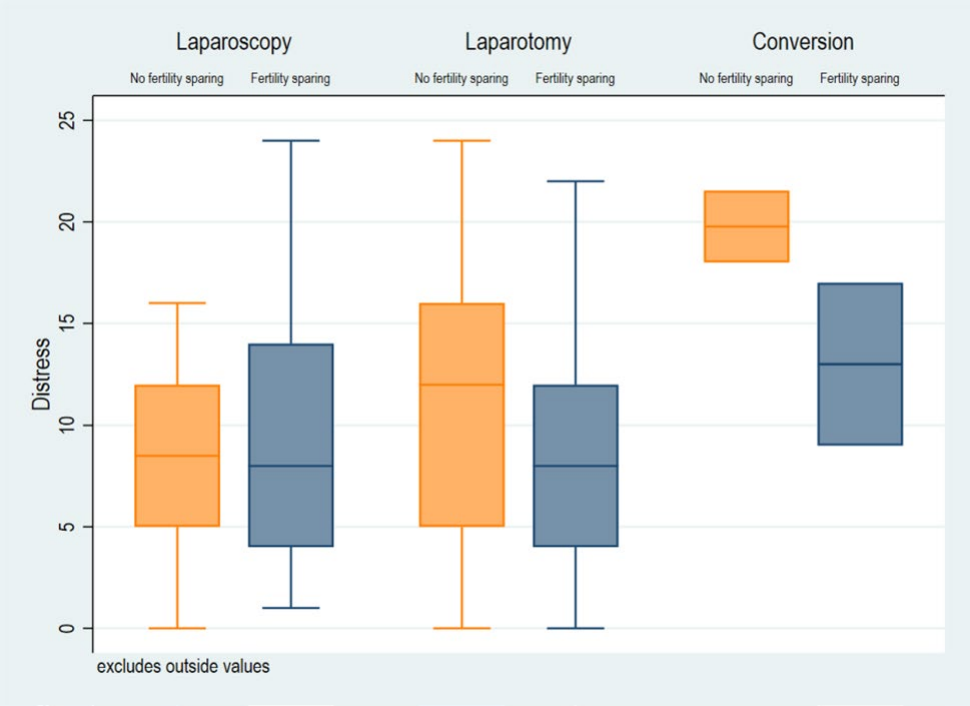
Boxplot of the level of distress in relation to the surgical approach in MOGCT and SCST patients with or without fertility sparing surgery

**Fig. 2:**
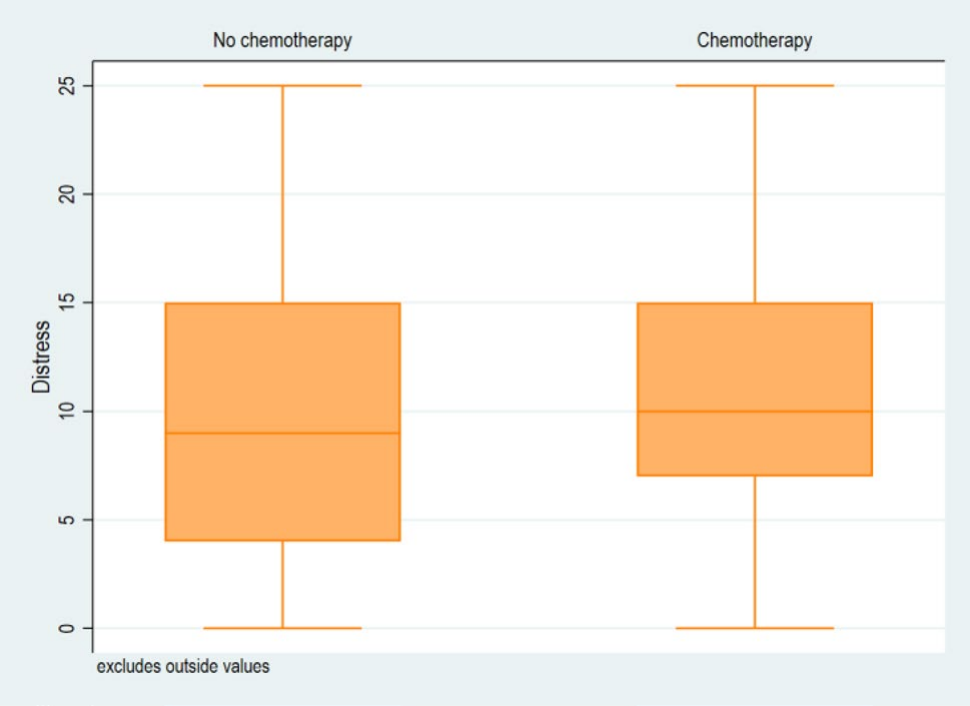
Boxplot of the level of distress in in MOGCT and SCST patients with or with or without adjuvant chemotherapy

When looking at the various potential predictors separately, there was no evidence for an association of neither the surgical approach nor whether fertility was preserved in primary surgery with distress in univariate analysis (Table 2). However, when investigating the predictors simultaneously in multivariate analysis, fertility sparing surgery was associated with lower levels of distress compared to non-fertility-conserving treatment (*β* = − 3.1, *p* = 0.04) (Fig. 1) in the total cohort. There was no evidence of association of administration of chemotherapy with distress levels (Fig. 2). Separating MOGCT and SCST patients in multivariate analysis, fertility-sparing surgery was not significantly associated with distress, nor with adjuvant chemotherapy, while controlling for age, recurrence and second tumor. This is probably due to the low sample size in the subgroups. Detailed data are shown in Table 3.

**Table 3:** Effect of fertility sparing surgery and chemotherapy on distress in subgroup analysis of MOGCT and SCST. Adjusted for age, recurrence and second tumor

MOGCT		Coef	*P-value*	Beta
Surgical technique	Non-fertility sparing	Ref		
	Fertility sparing	− 4.8	*0.15*	− 0.3
	Unknown	− 6.4	*0.19*	− 0.3
Adjuvant chemotherapy	No	Ref		
	Yes	3.1	*0.10*	0.3
SCST Surgical technique	Non-fertility sparing	Ref		
	Fertility sparing	− 3.5	*0.09*	− 0.3
	Unknown	3.4	*0.57*	0.1
Adjuvant chemotherapy	No	Ref		
	Yes	1.1	*0.56*	0.1
	Unknown	− 2.8	*0.76*	0.0

Mean pain level in the total cohort was 22.3, median 16.7. Pain was associated with an increase in distress. With every point on the pain scale, distress increased by 0.1 points. This was significant in univariate (*p* < 0.001,) and multivariate analysis (*p* < 0.001).

Women whose diagnosis dated back longer ago had no more or less distress than the ones with recent diagnoses (*β* = 0.1, *p* = 0.46). Similarly, there was no association whether the patient was living in a partnership or not to distress (*β* = 0.1, *p* = 0.92). Patients older than 70 years reported less distress (*β* = − 4.2, *p* = 0.04). (Table 2).

## Discussion

To the best of our knowledge, is this is the first publication of depression and anxiety in patients diagnosed with MOGCT or SCST. It is known that survival rates and life expectancy in patients with MOGCT and SCST are excellent, so that quality of life and the associated factors move into focus of survivorship [9, 11]. We showed that anxiety and depression are possibly influenceable long-term issues in some women with malignant germ cell tumors and analyzed associated factors in both tumor entities. Anxiety and depression are common psychological symptoms in cancer patients. The range of reported prevalence on anxiety and depression or distress in literature is wide due to various stage and type of cancer as well as numerous diagnostic criteria or tools. About one third of all cancer patients receiving oncological treatment and half of the patients who have advanced cancer suffer from mental distress to an extent that they can be diagnosed with a psychiatric disorder [15, 26]. In a longitudinal study on colorectal cancer patients, the prevalence rates for anxiety and depression is one in five with no significant change over time [27]. In breast cancer survivors, the prevalence of anxiety and depression after a median follow-up of 10 years is 20% [28]. There is no data on anxiety and depression in MOGCT or SCST so far. We showed that 34% of patients with MOGCT or SCST in our cohort had severe mental distress even after a median time of seven years after diagnosis. Psycho-oncological care should be offered to those patients. The level of distress was independent of the time passed since diagnosis.

Fertility-sparing surgery is currently the gold standard in MOGCT and SCST patients. It preserves the option for childbearing for the often young patients and hormone production is less altered. In a recent publication on MOGCT and SGCT patients, fertility-sparing surgery was associated with a significantly better global quality of life. The authors conclude that fertility preserving approaches should be offered to every MOGCT and SCST patient when acceptable from an oncologic perspective [29]. Cohen et al. reported that an induction of menopause in healthy women, non-fertility-conserving surgery or chemotherapy doubled the risk for the development of depression, indicating a correlation of depression and hormonal status (38). We showed that MOGCT and SCST patients who received fertility-sparing surgery had a lower risk for severe mental distress than those without fertility-conserving treatment, (*p* = 0.04) with a more significant effect in SCST patients (*p* = 0.09 vs *p* = 0.15) than in MOGCT. Merging these results with the conclusion of Hasenburg et al. [29] fertility sparing surgery results in a better quality of life and less severe mental distress. The underlying causes are unknown but the preservation of hormonal function and fertility may lead to less distress physiologically and psychologically. Hormonal substitution is not recommended in SCST patients, but can be considered in MOGCT patients [6, 30]. This might explain the lower incidence of severe mental distress in MOGCT patients, who did not receive fertility-conserving treatment.

Data from breast cancer patients show that a tumor recurrence does not lead to more distress in cancer survivors [31]. The authors hypothesized that coping phases might then be started over again not admitting anxiety to one self and only self-admitted anxiety is measured by HADS. In our cohort, neither tumor recurrence nor a second tumor led to an increase in depression and/or anxiety. The fear of disease recurrence might be a more relevant factor for the development of distress than the recurrence itself and should be addressed in psychological care after primary diagnose [16].

26% of long-term gynecologic cancer survivors suffer from pain. Pain has a negative effect on quality of life in these patients [32]. The absolute pain level in our patients was moderate with a median of 22.3/100. Interestingly with each point of rise in pain the level of mental distress did increase by 0.1 on HADS scale. Pain is a multidimensional phenomenon as a result of a complex interaction between physiological, psychological, cognitive, social, and other factors [33]. In our cohort a higher pain level correlated with an increase on the HADS scale, indicating that patients with higher pain levels were more likely to have relevant anxiety and depression levels. Yet it is unclear how the symptoms relate to each other. Do patients have more pain due to higher levels of anxiety and depression or does a higher level of pain cause anxiety and depression? The triggering factor should be treated with prioritization hopefully to prevent the correlating one. But if both symptoms correlate in a symptom cluster, treatment should target both symptoms, no matter which was diagnosed first. Spiegel et al. assessed pain and depression in two observational trials in cancer patients—most of them with metastatic breast cancer—and assumed that pain precedes depression, based on the observation that depression was more prevalent in the high pain group than in the low pain group of his trial [34]. Psychological distress is believed to have a direct physiological effect on sympathetic nervous system arousal, production of endogenous opioids, and level of muscle tension [35] possibly resulting in an increase in pain. A recent trial by Charalambous focused on the inter-relation and co-occurrence between those symptoms in patients with breast or prostate cancer. Aligning with our trial patients with increased pain were more likely to have higher levels of anxiety and depression. Parallel mediation analysis showed that anxiety, depression and fatigue fully mediated the relationship between pain and health-related quality of life (HRQoL), indicating that patients with increased pain are more likely to have higher levels of anxiety, fatigue and depression. The authors conclude, that pain needs to be targeted first, but anxiety and depression have to be addressed as well as they are essential targets for improvement of HRQoL in cancer patients [36]. As sufficient analgesia is one of the main topics in postsurgical care and well established, there is a need to further inform patients, physicians and caregivers about the relevance of sufficient analgesia in the survivorship period, also to possibly prevent anxiety and depression in long-term survivors of MOGCT and SCST. Pain is not only a physical experience but also involves various other components of human functioning including personality, mood, behavior, and social relations. A variety of psychological and cognitive behavioral treatments can reduce pain and should be offered to patients at a low threshold [22]. How do we identify those in need of help? Future incooperation of a routine screening of cancer patients during therapy as well as in short and long-term follow-up on depression, anxiety and pain is warranted. This is not only necessary because of its association with poorer HRQOL but also because of a higher all-cause mortality in cancer-survivors with depression [27, 37].

This analysis of CORSETT identifies long-term consequences for quality of life in survivors of MOGCT and SCST and highlights the necessity of screening on mental distress and pain not only in cancer patients but also in cancer survivors. Fertility preserving surgery should be offered to patients with MOGCT and SCST as it may help to improve well-being in cancer survivors.

The strength of our study is the large number of patients with a rare disease participating in the CORSETT and the detailed analysis of confounding factors in distress in these patients. As patients diagnosed with MOGCT or SCST within a period of ten years were asked retrospectively at one time point to fill out the questionnaires, there was a large range of time since diagnose and of the age of participants. This is a limitation of our trial as well as the low response rate. The high rate of recurrences in our cohort may be caused by the fact that healthy cancer survivors were more often lost to follow-up, presented less often in hospital and were less motivated to participate in trials of a survived disease. However, there has been little data on distress in MOGCT and SCST survivors highlighting the importance of our results.

## Data Availability

The datasets generated and analyzed during the current study are available from the corresponding authors on reasonable request.
